# The Effectiveness of a Tailored Faculty Development Program for Undergraduate Mentoring and Its Impact on Mentor’s Perceptions: A Mixed Methods Study

**DOI:** 10.7759/cureus.58863

**Published:** 2024-04-23

**Authors:** Smita Pakhmode, Yamini Pusdekar, Madhur Gupta, Anne Wilkinson, Satyadevi Uppu, Sheel Wasnik

**Affiliations:** 1 Department of Biochemistry, Narendra Kumar Prasadrao (NKP) Salve Institute of Medical Sciences and Research Center and Lata Mangeshkar Hospital, Nagpur, IND; 2 Department of Community Medicine, Narendra Kumar Prasadrao (NKP) Salve Institute of Medical Sciences and Research Center and Lata Mangeshkar Hospital, Nagpur, IND; 3 Department of Pathology, Narendra Kumar Prasadrao (NKP) Salve Institute of Medical Sciences and Research Center and Lata Mangeshkar Hospital, Nagpur, IND

**Keywords:** mentorship program, under graduate, medical, faculty development program, mentoring

## Abstract

Objective: “Anubandh” the existing mentorship program at our institute used to start with enthusiasm but lacked sustainability throughout the year. This study aimed to assess the need for designing and conducting a faculty development program (FDP) and evaluating its impact on effective mentoring.

Methodology: FDP was designed by assessing the perception of 50 teachers regarding undergraduate (UG) mentoring at a tertiary care teaching institute in central India, the NKP Salve Institute of Medical Sciences and Research Center and Lata Mangeshkar Hospital, Nagpur, India. It was developed and conducted by focusing on the global overview, mentoring policies of the institute and rationale while implementing mentorship. The effectiveness of FDP was tested by a change in pretest-posttest scores for assessing their knowledge and reflections on undergraduate mentoring.

Results: In the pre-FDP perceptions by mentors, the majority agreed that mentorship should be an integral part of the UG curriculum. Teachers felt that mentorship is an additional workload that needs to be given weightage in self-appraisal forms. A total of 81.2% of mentors emphasized on need for academic, social, and personal mentoring. After the FDP, there was a statistically significant improvement in the knowledge and attitude of mentors in all the assessed domains (χ^2^=2.648; df=6; p<0.05) like the need for UG mentoring in medical college and the role of faculties as a mentor. FDP sessions were appreciated by mentors for being motivating, interactive, and highly engaging with speakers having good oratory skills and using inspiring techniques with an overall rating of 9.2/10.

Conclusion: There was an overall positive attitude about mentoring but many expressed the need for training in mentorship. The FDP was highly effective in improving the knowledge and attitude of mentors for effective mentoring.

## Introduction

Mentoring is a global phenomenon happening in a wide range of fields apart from academics. The academic environment mostly has three levels where mentoring is observed - (a) between faculty and students who may be graduate or postgraduate students, (b) between senior and junior faculty, and (c) as per the hierarchy from dean to heads of departments on certain occasions [1]. Mentoring has become imperative with the advent of competency-based medical education in India. Furthermore, the concept has expanded above the academic domain to oversee even the emotional, mental, and psychological needs of the mentee [2,3]. The definition of the term “mentor” has therefore been refined surpassing the traditional role model to be defined as “a wise and trusted counselor or teacher” [4]. “Mentoring” is hereby referred to as the process by which an experienced person provides guidance, support, and encouragement to a less experienced person [5].

With the advent of competency-based medical education (CBME), it has become imperative to provide effective feedback to students for their improved learning and performance [6]. To meet this requirement several mentoring programs are being conducted at the institutional and university levels. However, there are no appropriate guidelines available for mentoring particularly regarding the content, processes, and structured mentoring protocols to be followed. Moreover, there is scarcity of data on the factors that can aid effective mentoring.

The existing mentorship program in our institute “Anubandh” was developed with an aim of providing effective mentoring to undergraduate medical students. The program encompasses assigning 10-12 students per mentor who meet periodically to discuss and develop solutions for issues ranging from academics to personality development, co-curricular activities, career plans, etc. Every year the program used to begin with enthusiasm from both the mentor and mentee sides but failed to sustain till the end of the academic session. First-year students used to attend the meetings frequently which gradually declined till the final year of Bachelor of Medicine and Bachelor of Surgery (MBBS) which is the short form coined from the Latin Medicinae Baccalaureus Baccalaureus Chirurgiae for the five-year undergraduate (UG) medical curriculum. In a few instances, teachers were not able to find time for meetings but most of the time students were not reporting for the meetings. With the passage of time motivation to attend and conduct meetings gradually declined. Hence, in order to evaluate the loopholes in the program and to make it more desirable for mentees as well as mentors in order to make it sustainable, the present study was designed. The study was also based on the idea of empowering the mentors for effective mentoring, therefore it became essential to study their perceptions regarding mentoring of the UG students. It was designed to assess the perceptions of mentors regarding the UG mentorship program followed by assessing the effectiveness of a faculty development program (FDP) that aimed to assess the mentor's knowledge of UG mentoring and their reflections on the FDP.

## Materials and methods

Study design, setting, duration, and population

This was a cross-sectional study using a mixed methods approach and a pre-post comparison of the mentor's knowledge regarding UG mentorship. The study was conducted at a tertiary care teaching institute, the Narendra Kumar Prasadrao (NKP) Salve Institute of Medical Sciences and Research Center and Lata Mangeshkar Hospital, Nagpur, in an urban area of Eastern Maharashtra. The study was conducted from September 2023 to December 2023. The study population was medical teachers who are involved in mentoring undergraduate students from all the years of medical school namely the per-clinical, para-clinical, and clinical departments of the institute. The following criteria were utilized for enrolling the study population.

Inclusion and exclusion criteria

All medical teachers involved in mentoring the students through the “Anubandh” program were invited to participate in the study and those consenting to participate in the FDP were included in the study. There were no pre-specified exclusion criteria.

Sampling technique and sample size

A convenience sample of 50 medical teachers from 168 teachers involved in teaching the medical undergraduates from the pre-clinical, para-clinical, and clinical subjects was taken, ensuring approximately equal representation from these subjects. No formal sample size was calculated. A sample of 50 teachers working as medical teachers in all four years of MBBS in the institute, Narendra Kumar Prasadrao Salve Institute of Medical Sciences and Research Center (NKPSIMS and RC), Nagpur, was selected as study participants by invitation and consent.

Ethical considerations and study procedures

The study was approved by the institutional ethics committee. The study was conducted in a step-wise manner with the first step being the assessment of the perceptions of all the teachers towards mentorship. This was done by developing and pre-testing a validated Google Form (Menlo Park, CA: Google LLC) that was shared with all the participating mentors. Their perceptions regarding the necessity of mentoring the UG students, attributes of an effective mentor and mentee, and what are the perceived barriers or facilitators for mentoring the UG students were assessed. Their perceptions about UG mentoring along with the preferred method for conducting meetings, source documentation, and the need for further capacity building for effective mentorship in the form of a faculty development program (FDP) for mentors were also assessed. The questionnaire comprised 20 items that included open and close-ended questions and “yes, no, maybe” response-type questions. Analysis of the perception of teachers was taken as a basis for developing the faculty development program for mentors. The findings of step one were incorporated for fine-tuning and designing a full-day FDP by the medical education unit of the institute.

The next step in the study was the designing and conduction of the FDP. After analyzing the responses of all mentors, it was found that most of the faculty members were aware of the basic aspects of mentoring. However, many of them required additional motivation, such as including weightage in self-appraisal, to actively participate in the mentorship program. Hence need for a faculty development program was felt so that all mentors should be trained and motivated to conduct the program with dedication. All investigators brainstormed for identifying topics and designs to be included in FDP. Faculties were identified from MEU to conduct the session. Pre-test, post-test, and reflection were designed on Google Forms.

One full day (6 hours) session was designed as a faculty development program. Need-based sessions were designed. A pre-test was conducted at the start of FDP. It started with the introduction of a mentorship program at the institute. It was followed by overview and perspective of mentoring programs in medical schools worldwide. In this session focus of mentorship in medical college like academic advancement, research guidance, career choice, career satisfaction, and work-life balance were explained to mentors. Measures to create and strengthen mentor-mentee bonds were also explained. In an interactive workshop session, the qualities of mentors and mentees were discussed, different situations and challenges were presented through role plays, and solutions were discussed. Finally, the mentorship policies of the institute were reinforced. The session concluded with a post-test and reflections of participants on FDP.

**Figure 1:**
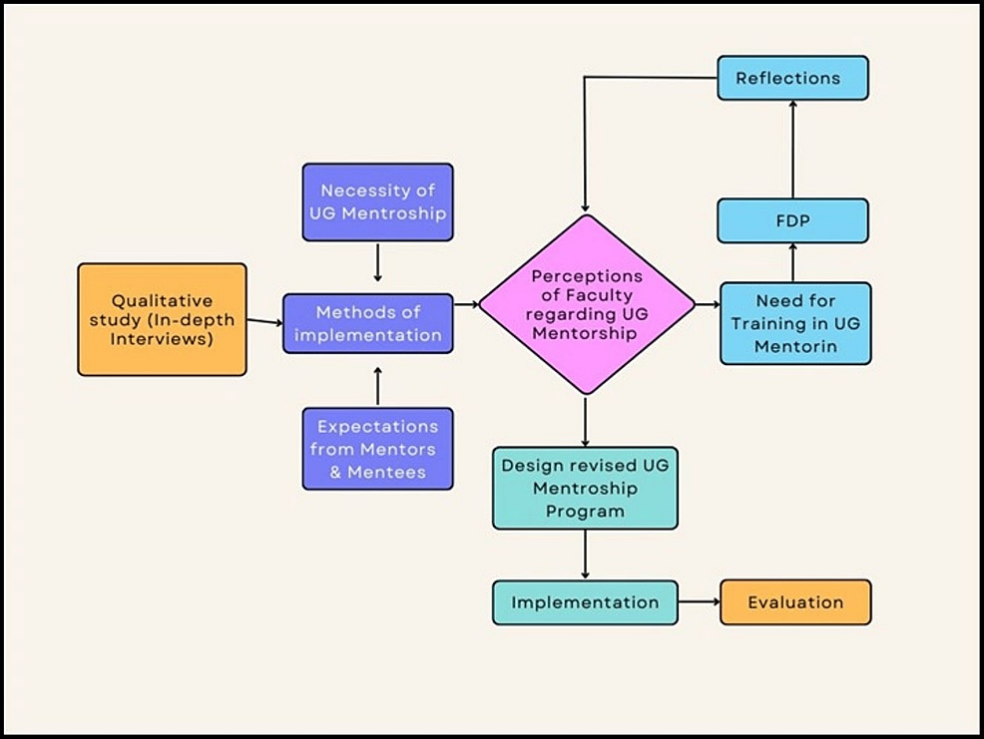
Flow diagram showing the study phases.

The FDP started with a pretest followed by a session to introduce the “Anubandh” mentorship program at the institute. The worldwide overview and perception of mentorship and mentoring policy at NKP SIMS and RC were covered in the next session. The rationale while implementing mentorship was activity-based workshop where roles of mentors, strategies to strengthen the mentor-mentee bond, and solution to problems during the implementation of mentorship program was offered by interactive and motivational techniques. Post-test was conducted at the end of the workshop. The third step in the study entailed the collection of the reflections of faculties regarding mentorship issues and the conduct of the faculty development program overall. Pre-test, post-test, reflection for the program were conducted online using Google Forms. The conceptual framework for the study is illustrated in Figure 1.

Statistical analysis

**Figure 2:**
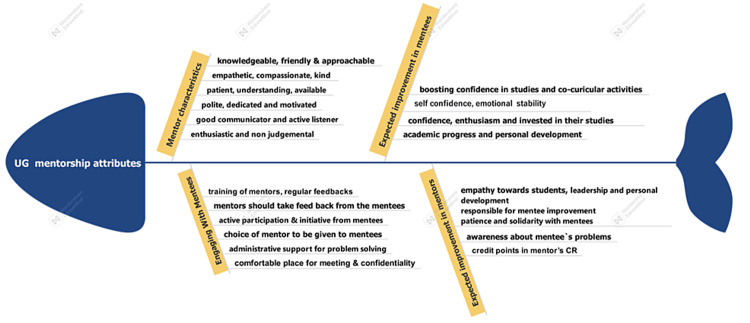
Fishbone diagram showing perceptions on UG mentorship (n=49). UG: undergraduate

The sociodemographic data of the participants including their age, educational qualifications, gender, and years of experience as faculty and as a mentor were entered in MS Excel (Redmond, WA: Microsoft Corp.). Percentages, proportions along with mean and standard deviations were calculated for the pertinent sociodemographic variables. Their perceptions about the mentorship program were recorded through Google Forms and analyzed using the perceptual analysis approach that focuses on the perceptual understanding of the facts as stated by the participants. This helps in delineating the subjective opinions of the participants about a particular behavior, experience, or phenomenon without judging them as appropriate or inappropriate. A fishbone diagrammatic representation of the perceptions in different areas like mentor characteristics, factors for engaging mentees as well as expected changes in the mentor and mentees through effective implementation of the mentorship program (Figure 2). The broader perceptions were grouped in the above-mentioned categories using the grounded theory approach for qualitative data analysis. The improvement in knowledge regarding UG mentoring after conducting the FDP was assessed using the pre-post test scores by analyzing the pre-test-post-test score difference method, or the gain in scores after attending the FDP. In this analysis, the data were simplified by transforming the bivariate pre-test and post-test values into univariate values using the difference between the post-test and pre-test scores and by analyzing the difference in the scores using the Chi-square test. A p-value of <0.05 was considered statistically significant. The reflections about the FDP were analyzed using thematic analysis conducted to induct the themes related to the reflections regarding what was good about the FDP along with the possible areas for improvement in the FDP. Free coding of the qualitative data for reflections about the FDP was done manually using the phrases and keywords used by the participants to assign codes to them. After coding the data, the independent or recurring themes that emerged within the data were identified for the thematic analysis. This description has now been added to the statistical analysis section. The thematic analysis was conducted on the lines of Braun and Clarke’s six-phase framework using the following steps: step 1, becoming familiar with data; step 2, initial codes generation; step 3, searching for themes; step 4, reviewing and grouping similar themes; step 5, defining themes; step 6, write-up the themes emerging from the overall dataset [7]. The feedback on the FDP was devised into a rating out of 10 points in the pertinent areas of the FDP.

The FDP Rating scale developed for the purpose of the study for assessing the effectiveness of the FDP comprised four main domains as shown in Table 1. Each domain had items scored from 0-3 or 0-2 and an overall score of 10 was allocated to the rating scale with a score of >9 as excellent, 7-9 as very good, 5-7 as good score, and >5 as a poor score.

**Table 1:** Scale for assessment of faculty development program in different domains. The scale was developed by the author (Yamini Pusdekar) of this study.

Domain	Score
Ease of understanding	Easy: 3, difficult: 2, very difficult: 1, unable to understand: 0
Language	Simple: 2, complex: 1, very complex: 0
Skill development	Good: 2, fair: 1, poor: 0
Willingness to recommend FDP to fellow faculties/mentors	Very likely: 3, likely: 2, less likely: 1, never: 0

## Results

This cross-sectional, mixed methods study was conducted to assess the perceptions of medical faculties on UG mentorship followed by developing and conducting an FDP. The perceptions of the teachers and their pre-post scores were analyzed to evaluate its impact on UG mentorship and their reflections about the FDP were obtained. A total of 50 faculties were invited to participate, from which one of the forms was incomplete and was therefore excluded from the analysis giving a total sample of 49 and a response rate of 98%.

The participating faculties were approximately equally distributed among the pre-, para-, and clinical subjects with the majority 18 (36.7%) being from the clinical departments. Most of the participants 23 (46.9%) were from the age group of 35-45 years with ages ranging from 25-63 years, mean age was 37.5 (SD: 7.32) years. There was a female preponderance, with nearly two-thirds (33 {67.4%}) of them being females. A total of 39 (75.5%) of the teachers participated in the UG mentorship program and 24 (61.6%) had participated for more than a year (Table 2).

**Table 2:** Baseline characteristics of the study population (n=49). *One of the forms was incomplete, with missing data on the pertinent variables of interest. Hence, it was excluded from the analysis, giving a response rate of 98.0%. **Eleven participants had not participated in the UG mentorship program hence the data for 39 participants is provided. UG: undergraduate

Characteristics	Number	Percentage
Department (n=49)*		
Pre-clinical	15	30.6
Para-clinical	16	32.7
Clinical	18	36.7
Age (in years) (n=49)*		
25-35	5	10.2
35-45	23	46.9
45-55	14	28.6
55-65	7	14.3
Gender (n=49)*		
Male	16	32.6
Female	33	67.4
Participation in UG mentorship program (n=49)*		
Yes	39	75.5
No	10	24.5
Years of participation in UG mentorship program (n=39)**		
1-3 years	24	61.6
>3 years	9	23.0
Can’t remember	6	15.4

**Figure 3:**
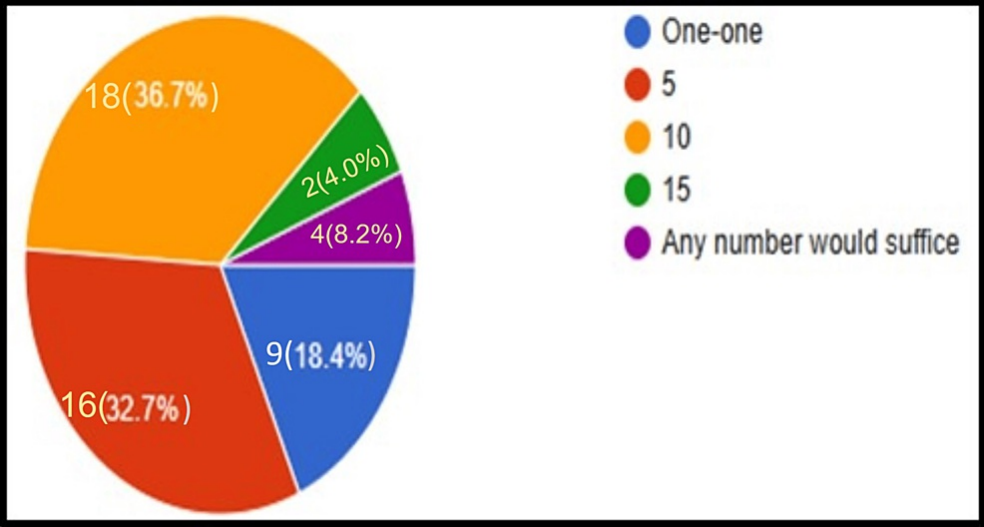
Mentor's perception regarding the number of mentees that each mentor can be assigned (n=49).

**Figure 4:**
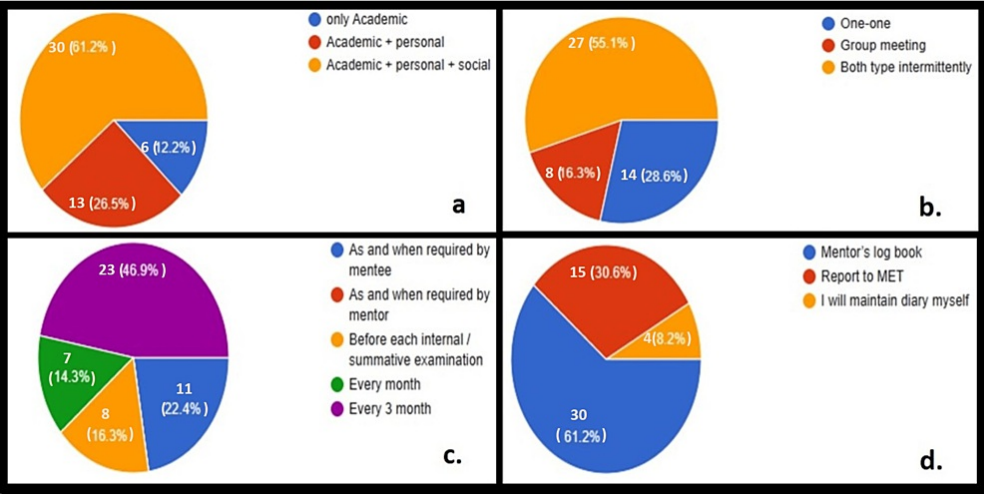
Perception of mentors regarding meeting conditions for mentoring UG students with respect to (a) type of meeting, (b) mode of meeting, (c) frequency of meeting, and (d) suitable method to record the meeting (n=49). UG: undergraduate

When asked about the optimal number of students assigned per mentor, the majority 69.4% of the mentors believed that five or 10 students should be allotted to each mentor for effective mentoring (Figure 3). When their perceptions regarding ideal meeting conditions for effective mentoring of the UG students with respect to (a) type of meeting, (b) mode of meeting, (c) frequency of meeting, and (d) suitable method to record the meeting were enquired, it was stated by 61.2% of the mentors that rather than focusing only on academics the meetings should target academic, personal and social aspects of the mentee's life. The commonest mode of meeting was stated to be a combination of one-on-one and group meetings conducted intermittently as mentioned by 55.1% of the mentors. The majority of the mentors (46.9%) stated that the meetings should be conducted on a quarterly basis, whereas nearly 22.4% of them believed that the meetings should be conducted as and when required based on the student's and mentor's requirements. The mentor's log book was the most commonly stated method for documentation of the meetings by 61.2% of the participants (Figures 4a-4d).

In the pre-FDP perceptions by mentors, the majority agreed that mentorship should be an integral part of the UG curriculum. Teachers felt that mentorship is an additional workload that needs to be given weightage in self-appraisal forms (Table 3).

**Table 3:** Perceptions of faculties about UG mentorship program (n=49). UG: undergraduate

S. no.	Questions	Yes, n (%)	No, n (%)	Maybe, n (%)
Perceptions of faculties regarding UG mentorship				
1.	Do you think mentoring is an integral part of the UG teaching program?	40 (81.6)	0 (0)	09 (18.4)
2.	Do you think UG mentorship is needed for UG medical students?	46 (93.9)	2 (4.1)	1 (2.0)
3.	Do you think every student should be included in the program?	31 (63.2)	18 (36.8)	0 (0)
Attitudes of mentors toward UG mentorship				
1.	Would you like to be a part of the UG mentoring program?	39 (79.6)	2 (4.1)	08 (16.3)
2.	Do you feel mentoring is an additional workload for medical teachers?	15 (30.6)	24 (49)	10 (20.4)
3.	Do you feel mentors should be given additional marks in self-appraisal form for the annual confidential report of an individual teacher?	46 (93.9)	2 (4.1)	1 (2.0)
4.	Would you like to have a student mentee from the same MBBS year where you are teaching?	1 (63.2)	18 (36.8)	0 (0)

The perceptions of the faculties with regards to what constitutes effective mentorship were asked, it was mentioned that several mentor characteristics like knowledge about the subject, friendliness, compassion, kindness, understanding, dedication, and motivation were of utmost importance when preparing to be an effective mentor. The teachers also mentioned that the mentor should be polite, receptive, non-judgmental, an active listener, and have good communication skills. The mentors also expressed the need for getting trained in mentorship as a part of their job profile as it will help them in implementing the mentorship program effectively for the benefit of the students at large. These features will help them to connect well with the mentees and the feedback given to them is well received and the mentees feel comfortable to open up to such mentors. Several factors were found to be important for engaging mentees like regular feedback from mentees and ensuring active participation of mentees by offering them the choice to select their preferred mentor. The role of administration was also emphasized by providing a comfortable place for arranging the meetings and solving conflicts if any in addition to maintaining strict confidentiality.

The effective implementation of the mentorship program is expected to bring about certain benefits for the mentors along with several benefits for the mentees. The mentees are expected to become more self-confident both in their studies and co-curricular activities. The mentorship sessions also boost the emotional stability of the mentees ensuring their academic progress as well as overall personality development. The mentors also benefitted during the process, as they developed an empathetic and responsible attitude towards their mentees. Their leadership qualities are also honed during the processes while developing solidarity with the mentees through becoming aware of their problems and devising practical solutions for the same. The mentors also anticipated that mentoring should become a way to earn brownie points in their confidential reports if taken up in addition to their routine activities (Figure 2).

Table 4 shows the improvement in the knowledge of mentors after implementing the FDP. It was observed that after implementing the FDP, there was a statistically significant improvement in the knowledge and attitude of mentors in all the assessed domains (χ^2^=2.648; df=6; p<0.05) like the need for UG mentoring in medical college and the role of faculties as a mentor.

**Table 4:** Effectiveness of the FDP for UG mentoring (n=49). *Multiple responses per person. **Chi-square (χ^2^) test is applied to the overall knowledge improvement and is statistically significant (p<0.05) (χ^2^=2.648; df=6). FDP: faculty development program; UG: undergraduate

Components of UG mentorship*	Correct knowledge about UG mentoring				p-Value**
	Pre-FDP		Post-FDP		
	Number	Percentage	Number	Percentage	
Need of UG mentoring in medical institutions	7	14.3	34	69.3	<0.0000001
Role of faculties as mentor	11	22.4	41	83.6	<0.0000001
Qualities of mentees	10	20.4	44	89.8	<0.0000001
Difficulties in implementing UG mentorship program	18	36.7	36	73.5	0.00003310
Strengths of UG mentorship programs	11	22.4	38	77.5	<0.0000001
Suggested improvements in mentoring program	16	32.6	45	91.8	<0.0000001
Overall	23	46.9	47	95.9	<0.0000001

**Figure 5:**
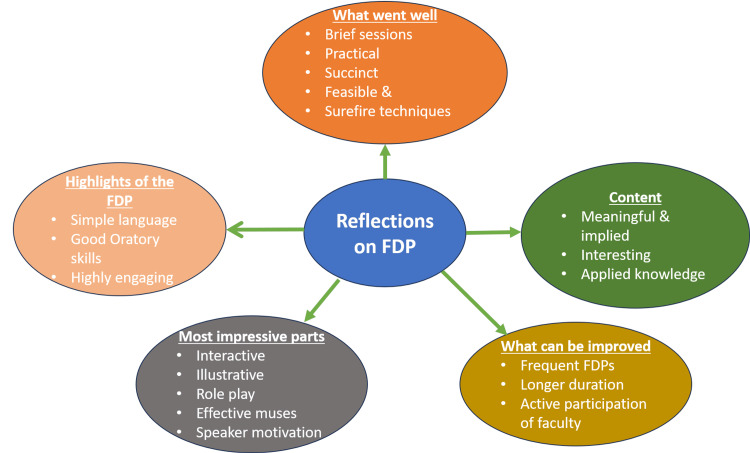
Reflections of the faculty about the FDP (n=49). FDP: faculty development program

Figure 5 shows the reflections of the mentors on the FDP. They mentioned that the sessions were conducted in simple language and the speakers had good oratory skills. The FDP sessions were appreciated by mentors for being motivating, interactive, and highly engaging. The speakers were noted for having good oratory skills and using inspiring techniques with an overall rating of 9.2/10.

The thematic analysis of the reflections revealed the following four thematic areas: (i) highlights of the FDP, (ii) engaging parts of the FDP, (iii) content as the strength, and (iv) suggestions for further improvement. These thematic areas are described below.

Highlights of the FDP

The mentors who attended the FDP mentioned that the highlights of the training were that the sessions were brief and hence engaging. The mentoring techniques taught during the one-day program were succinct, of practical utility, feasible, and based on surefire techniques that could be used in day-to-day life. This was expressed through the following quotes. “In olden days there was no special mentoring needed as the students were very close to teachers and would turn up on their own to discuss their difficulties. The training shed light on effective techniques for engaging mentees by using effective feedback techniques” - senior faculty. “The FDP was an eye opener to us who are already engaged in mentoring MBBS students as the techniques taught are surefire and immensely useful as practical pointers for effective UG mentoring” - junior faculty who is also a mentor.

Engaging parts of the FDP

The other important reflections about the FDP were that the illustrations used for teaching about effective mentoring as well as the interactive techniques used like role play were beneficial as effective muses. Moreover, the motivation of the speakers had a positive impact on the resolve of the mentors to effectively take the “Anubandh” program to new heights. This was explicitly illustrated in the below-mentioned supporting quotes. “The role play on good and bad mentor made me introspect particularly on the soft skills that are useful in mentoring students” - junior faculty and mentor. “Several illustrations of each of the mentor qualities was an effective muse that could be directly adapted in our mentoring regimes” - senior faculty and mentor. “The speakers themselves were highly motivated and set an effective example as to how mentoring can bring about positive impact in the lives of our mentees as well as ourselves which I found very inspiring” - senior faculty. “Kudos to the entire team of the medical education unit for creating this program that will definitely create a great task force for mentoring the upcoming doctors” - senior faculty and mentor.

Content as the strength

The mentors mentioned that the content of the FDP was meaningful, implied, interesting, and focused on applied knowledge. The expressions of the participants that support the above statement have been described below. “The content was meaningful and highly engaging that made me reflect on the current mentoring practices and the dire need to revamp these” - senior faculty. “Concepts taught in the FDP had great practical applicability” - junior faculty. “Interesting anecdotes mentioned by the speakers with real-life experiences while mentoring gave an implied meaning to the FDP” - junior faculty and mentor. “The contents of the FDP were tailored to the mentoring needs of both mentors and mentees” - senior faculty. “The sessions were thoroughly planned and adapted to the mentor perspective” - senior faculty and mentor.

Suggestions for further improvement

When asked about the facets of the FDP that could be improved further, the mentors mentioned that conducting the FDP more frequently and for more duration, i.e., one-day sessions and one-day hands-on activities will ensure active participation by mentors. “Would it have been a two-day affair with hands-on skill development added to it, it would have been a tremendous treat for junior faculties like us who have just begun as mentors” - junior faculty. “Such programs should be conducted frequently to hone and update our mentoring skills” - junior faculty.

## Discussion

The study aimed to assess the perceptions of faculties as mentors in order to determine the gaps in mentoring that can be used to design a tailored FDP for mentors focussing on effective mentoring techniques. The pre-post scores of the mentors regarding their knowledge about mentoring were also assessed. There was a significant improvement in the knowledge of teachers about effective mentoring techniques. 

The CBME has brought in its wake several challenges in mentoring students in ways that are student-centered and bring about all-around development of mentees. The benefits of mentorship not only go beyond traditional academic performance but also encompass emotional and personal aspects, including but not limited to career development, interpersonal relationships, research interests, career aspirations, improved self-esteem, and stress-free student life [7,8].

Though teaching takes place in most of the medical institutes, mentoring is not done at all, or even if done, it is unstructured and without any guided protocols for the same. In our institute, though there was an established protocol and a structured mentorship program in the form of “Anubandh,” still most of the mentors both junior and experienced expressed the need for further guidance on effective mentoring techniques. This was also elaborated by other studies that have tried to delineate the process of effective mentoring. It is considered to be an insightful process of acquiring the mentor's wisdom by the mentee and its application after modification as needed by the mentee. The mentoring process is akin to teaching in many ways but is additionally supportive and protective towards the mentee to bring about their overall development [9].

Though the mentorship program was acknowledged to be an important aspect of student's advancement and a key to their success, still there were conflicts in opinions on the processes to be followed during an effective mentoring session. The opinions regarding whether the meetings should be conducted online or offline, their frequency, maintenance of meeting minutes and logs, etc., were debatable. Other studies have also reported a lack of consensus on effective mentoring processes [10,11]. This necessitates the development of policies for mentoring at the institutional level that would prove beneficial to both the mentors and the mentees [12,13].

The present study reported that the mentor plays several roles as a counselor, friend, advisor, and guide for pertinent academic and other choices that the mentees make during their life course. This was similarly reported by a study conducted by Swe and Bhardwaj where the participating mentors reported the most common role as counselor among nearly 40%, as a career guide (22.91%), perceived as a role model by 16.6%, while 8.3% defined mentor as a research guide [14]. In yet another study by Lian et al., the mentors perceived that mentoring is all about counseling, as it offers the best method for understanding and solving mentee problems [15].

Unlike teaching or tutoring the students, mentors are supposed to actively help the mentees to reach their full potential in order to achieve their personal as well as professional goals [12]. This poses an additional burden on the already burnt-out medical teachers who have to multitask with their clinical, teaching, and administrative roles leaving very little time to cater to mentoring [13]. The provision of incentives in the form of credit points in their appraisal forms could be a motivator for teachers to pursue mentoring with vigour bringing about the desired results for the mentees as well.

The study reports several benefits including increased self-confidence, academic performance, and emotional stability for the mentees through the mentorship program. Similarly, the mentors benefitted by developing leadership skills, being acquainted with the problems of their students, and a sense of fulfillment by empathetically resolving their mentee's problems. This is also reflected in the existing research by identifying the perceived benefits of mentoring by both mentors and mentees. Mentors benefitted from an improved ability to reflect and being able to solve problems. Mentors have also reported better job satisfaction and academic recognition [14,15]. Mentees considered one-to-one mentorship as the most effective method for effective mentoring programs. The mentor acted as both a professional and personal role model, as well as a career counselor, helping mentees make early and effective specialty and career choices. Mentees expressed that mentorship experiences aided in improving their knowledge and performance and provided great emotional support at times of stress [16-21].

Several previously conducted studies on the effectiveness of mentoring programs have shown results that are concordant with our present study. According to a study conducted by Levinson et al., a mentorship program was instrumental in enhancing academic productivity, career advancement, and satisfaction among mentees [17]. Applegate and Williams showed that mentoring positively impacts personal and professional behaviors of mentees [18]. Keating et al. also showed that mentoring brings about a positive impact, especially among at-risk youth [19]. Erickson et al. emphasized the impact brought about by mentors on the overall academic achievement of the mentees [20]. Hawkins et al. also reflected on the key benefits of mentorship as improved academic record, enhanced self-esteem, and confidence among the mentees [22].

A study by Jayalakshmi et al. also observed that mentoring helps to build a friendly relationship with mentees that creates a relaxed environment aiding effective communication and better understanding of each other [21]. The mentors also opined that mentoring the students from the same year as the teacher would be effective. There was a common consensus on the frequency, mode, and type of meeting in our study with most mentors favoring both face-to-face and online modes and frequency as desired by the mentees. Lian et al. mentioned that the frequency of meetings depended on the time and availability of both mentors and mentees. Thus, biannual meetings that coincide with academic results are more suitable [15].

In the present study, the mentors expressed a need for FDP on mentoring that was designed and implemented after obtaining the initial perceptions of mentors. Post-test scores after conducting the FDP revealed a statistically significant improvement in the knowledge about mentorship. In the study by Swe and Bhardwaj similar opinion was expressed by the mentors that they should undergo formal training before or in service as a medical teacher [14]. The training would prove beneficial for skill development for better communication and problem-solving. They also stated that the training would serve as a guideline for effectively conducting the mentorship program [23-25]. The FDP was well received by the mentors and appreciated for being conducted in simple language and the speakers had good oratory skills. The FDP sessions were appreciated by mentors for being motivating, interactive, and highly engaging with speakers having good oratory skills and using inspiring techniques with an overall rating of 9.2/10.

Strengths

One of the strengths of using the mixed methods approach is the relative advantage offered by the qualitative component of being able to assess in-depth perceptions on a topic about which little is known among the participants. The additional use of quantitative methods adds to the robustness of the qualitative component by according verification of the facts mentioned by individuals and also at times helps in generalizing the findings obtained through the qualitative study.

Limitations

A convenience sample from a single institute limits the generalizability of the results. The sample was selected because of its accessibility to the researchers. However, the results were beneficial at the institutional level for recommending reforms such as pre-recruitment or in-service FDP on mentoring as a norm. Another limitation may be the avoidance of selection of extreme response categories for their responses on perceptions regarding mentoring which may have led to central tendency bias. However, this was minimized by the use of concurrent open-ended questions that elaborated the perceptions further.

Implications in practice

One of the successes of the research was the establishment of regular FDP sessions on effective mentorship. Future implications of the study include developing key resources on mentoring techniques that could be utilized by other underprivileged institutions where mentoring support systems do not exist for guiding their mentors or if required could be extended to their mentees too. Thereby mentors will be empowered to conduct effective mentorship programs, have empathy towards mentees and achieve greater job satisfaction, improve their leadership skills, instructional capacity, and, also positively impact student performance. However, continuous quality monitoring and evaluation of the mentoring processes as well as mentor-mentee relationships would provide much-needed supportive supervision to the mentors for achieving higher mentee satisfaction and their overall development in all academic and co-curricular spheres.

## Conclusions

There was an overall positive attitude about mentoring but many expressed the need for training in mentorship. The FDP on mentoring techniques was highly effective in improving the knowledge and attitude of mentors for effective mentoring. The medical education unit at the participating institute has now established a faculty development program on mentoring on an annual basis.
